# Complicated Postoperative Flat Back Deformity Correction With the Aid of Virtual and 3D Printed Anatomical Models: Case Report

**DOI:** 10.3389/fsurg.2021.662919

**Published:** 2021-05-28

**Authors:** Jennifer Fayad, Mate Turbucz, Benjamin Hajnal, Ferenc Bereczki, Marton Bartos, Andras Bank, Aron Lazary, Peter Endre Eltes

**Affiliations:** ^1^In Silico Biomechanics Laboratory, National Center for Spinal Disorders, Buda Health Center, Budapest, Hungary; ^2^Department of Industrial Engineering, Alma Mater Studiorum, Universita di Bologna, Bologna, Italy; ^3^Department of Spine Surgery, Semmelweis University, Budapest, Hungary; ^4^School of PhD Studies, Semmelweis University, Budapest, Hungary; ^5^Do3D Innovations Ltd., Budapest, Hungary; ^6^National Center for Spinal Disorders, Buda Health Center, Budapest, Hungary

**Keywords:** 3D printed anatomical models, flat back deformity, 3D virtual model, 3DPDF, fused deposition modeling

## Abstract

**Introduction:** The number of patients with iatrogenic spinal deformities is increasing due to the increase in instrumented spinal surgeries globally. Correcting a deformity could be challenging due to the complex anatomical and geometrical irregularities caused by previous surgeries and spine degeneration. Virtual and 3D printed models have the potential to illuminate the unique and complex anatomical-geometrical problems found in these patients.

**Case Presentation:** We present a case report with 6-months follow-up (FU) of a 71 year old female patient with severe sagittal and coronal malalignment due to repetitive discectomy, decompression, laminectomy, and stabilization surgeries over the last 39 years. The patient suffered from severe low back pain (VAS = 9, ODI = 80). Deformity correction by performing asymmetric 3-column pedicle subtraction osteotomy (PSO) and stabilization were decided as the required surgical treatment. To better understand the complex anatomical condition, a patient-specific virtual geometry was defined by segmentation based on the preoperative CT. The geometrical accuracy was tested using the Dice Similarity Index (DSI). A complex 3D virtual plan was created for the surgery from the segmented geometry in addition to a 3D printed model.

**Discussion:** The segmentation process provided a highly accurate geometry (L1 to S2) with a DSI value of 0.92. The virtual model was shared in the internal clinical database in 3DPDF format. The printed physical model was used in the preoperative planning phase, patient education/communication and during the surgery. The surgery was performed successfully, and no complications were registered. The measured change in the sagittal vertical axis was 7 cm, in the coronal plane the distance between the C7 plumb line and the central sacral vertical line was reduced by 4 cm. A 30° correction was achieved for the lumbar lordosis due to the PSO at the L4 vertebra. The patient ODI was reduced to 20 points at the 6-months FU.

**Conclusions:** The printed physical model was considered advantageous by the surgical team in the pre-surgical phase and during the surgery as well. The model was able to simplify the geometrical problems and potentially improve the outcome of the surgery by preventing complications and reducing surgical time.

## Introduction

As the number of instrumented spinal operations increases globally, the group of patients with iatrogenic spinal deformities is growing (1). Loss of lordosis, development of segmental or global kyphosis after a shorter or longer thoracolumbar stabilization are the most common form of iatrogenic (so called “flat back”) deformities (2). Beyond the consequent spinal canal stenosis, the disturbance of global balance can result in severe disability and pain where only surgical correction of the spinal alignment can provide significant functional improvement (2). The common sagittal balance problem in some cases is complicated with coronal imbalance, making the surgical correction procedure more complex. Further anatomical and geometrical irregularities caused by the previous surgeries (e.g., lack of anatomical landmarks, segmental bony deformations) makes the situation more challenging. In such cases, meticulous preoperative planning and proper implementation of the surgical plan are the keys to success and advanced scientific tools are needed to support the process and improve the outcome.

Here, we present the case of an elderly female patient with severe sagittal and coronal malalignment due to repetitive spine surgical interventions for over 39 years. Virtual and 3D printed patient-specific models were used to understand the unique and complex anatomical-geometrical problem and to plan the proper surgical correction.

## Case Presentation

### Medical History

A 71-year-old female patient was admitted to our institution. She suffered from severe low back pain, irradiating to the left leg, and an inability to walk more than 50 m due to fatigue in both lower extremities. There were some significant, treated comorbidities in her medical history: chronic hypertension, non-insulin-dependent (type II) diabetes, and ischemic heart disease. Previously, the patient's back problems were treated in other hospitals. The first discectomy surgery at the level of L4/S1 was performed 39 years ago, since then a mild L5 sensory-motor deficit persisted on the right side. Sixteen years later, L4/5 discectomy was performed followed by an L3/4 discectomy a year later. A repeated discectomy was done at L4/5 level 4 years ago, followed by a discectomy/decompression at the L2/3 level due to signs of cauda syndrome. The last surgical intervention in another hospital was done a year later (3 years ago), when an L2-L4 posterior stabilization and L3 laminectomy without intervertebral fusion was performed.

### Evaluation and Analysis

Physical examination showed severe sagittal and coronal imbalance, compromised gait, tenderness at the lower back area and spastic muscles. She suffered from mild distal motor weakness in both lower extremities and numbness of the left leg. Based on her examination and imaging studies [full spine X-ray, lumbar CT (Supplementary Figure 1) and MRI (Supplementary Figure 2)], the severe lumbar sagittal and coronal malalignment was identified as the primary source of pain. Beside the deformity, non-union and partial implant loosening at the L2-L4 segments, and degenerative instability at the L1/2 and L3/4 segments were diagnosed. Patient's pain was assessed by Visual Analog Scale (VAS = 9 preoperatively), and disability was measured using the Oswestry Disability Index (ODI = 80% preoperatively) (3). Surgical treatment was indicated considering the spinal pathology, severe pain, disability, and life quality deterioration.

### Analysis of Spinopelvic Alignment in Terms of Surgical Correction

**Figure 1:**
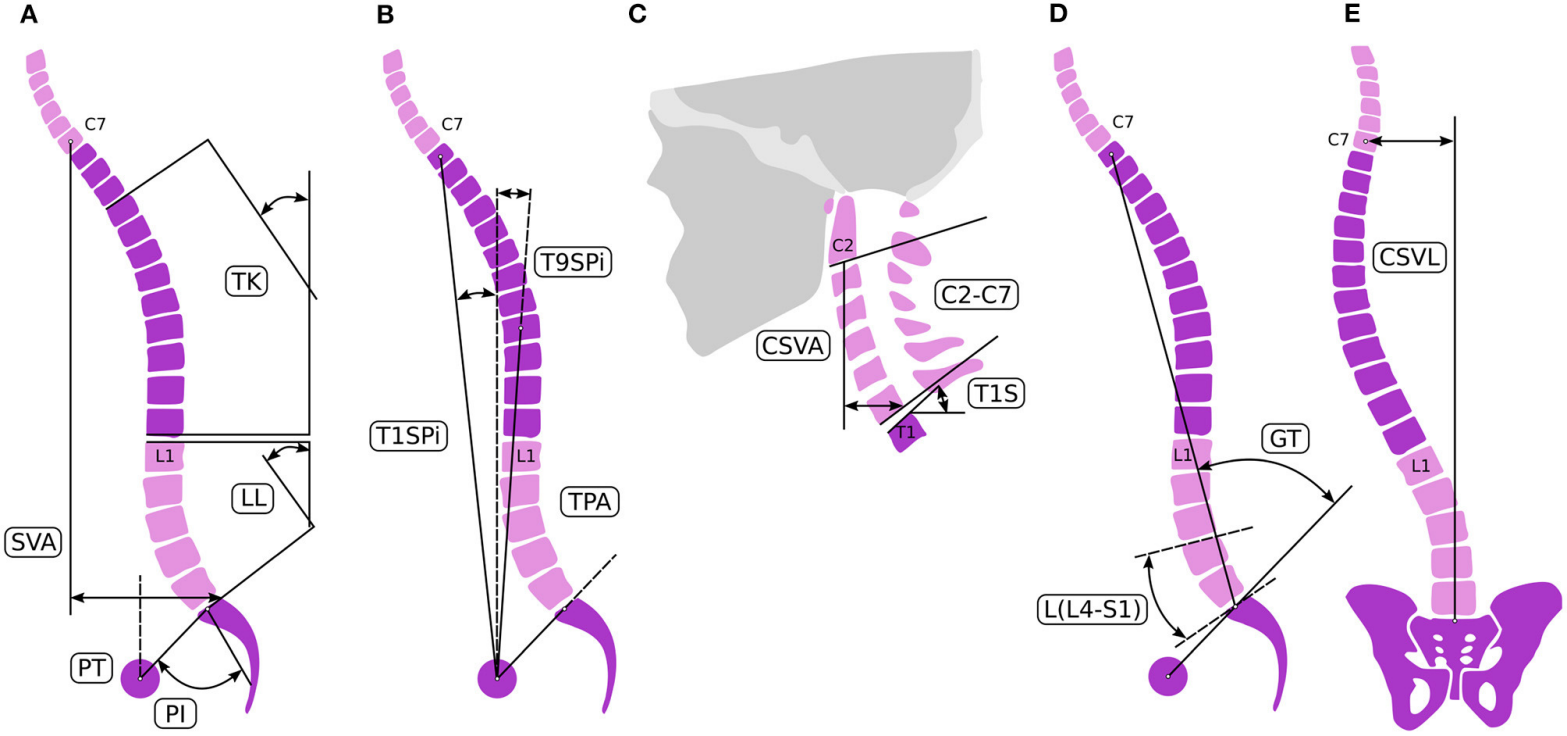
Spinal alignment evaluation. Sagittal spino-pelvic parameters **(A–C)** for the assessment of the alignment [**A–C** adopted from Lafage et al. (4)]: pelvic parameters measured were PI, PT, and SS. Regional spinal parameters included PI-LL mismatch, LL, and TK. Global alignment was assessed linearly by SVA and the angular measurements of T1SPI, T9SPI, and TPA. Cervical parameters were composed of T1 slope, C2–C7 cervical lordosis, and C2–C7 SVA. **(D)** For the GAP score L4-S1 lordosis L(L4-S1) and the global tilt (GT) were defined. **(E)** Coronal alignment is assessed by measuring the distance between the center of the C7 vertebral body and the CSVL.

Global balance and spinopelvic alignment were analyzed to determine the objective of the correction. Parameters describing the spinopelvic alignment were calculated from standing X-ray using the Surgimap software (Nemaris Inc., New York, NY, USA). Pelvic incidence (PI), pelvic tilt (PT), sacral slope (SS), lumbar lordosis (LL), and thoracic kyphosis (TK) were measured (4) (Figure 1). Global sagittal alignment parameters such as the sagittal vertical axis (SVA), T1 spinopelvic inclination (T1SPi), T9 spinopelvic inclination (T9SPi), and T1 pelvic angle (TPA) were also calculated (5). The Global Alignment and Proportion (GAP) Score was calculated according to the method published by Yilgor et al. (6). The coronal alignment was assessed by measuring the distance between the center of C7 vertebral body and the central sacral vertical line (CSVL) (7). The measurements are summarized in Table 1.

**Table 1:** Parameters for the evaluation of the spinal alignment pre- and postoperatively.

**Parameter**	**Preop**	**Postop**
PI (°)	55	55
PT (°)	27	21
SS (°)	28	34
LL (°)	17	47
PI-LL (°)	38	8
TL (°)	2	5
TK (°)	16	30
T9SPI (°)	0	6
T1SPI (°)	7	1
TPA (°)	34	22
T1S (°)	41	31
CL (°)	39	32
GT (°)	12	28
L(L4-S1) (°)	14	31
C2-C7 SVA (mm)	12	20
SVA C7-S1 (mm)	156	82
C7 to CSVL distance (mm)	48	5
GAP score	8	3

The central origin of the patient's complaint was the loss of lordosis at the lumbar spine due to the degenerative and iatrogenic processes. The patient's global balance was characterized as an imbalance both in the sagittal and coronal planes. The GAP score was 8 preoperatively, corresponding to severely disproportioned alignment. Therefore, the aim of the surgical correction was the 3D correction of the lumbar alignment. To calculate the degree of the desired lordosis correction, different approaches were sequentially applied. First, we used the formula published by Le Huec et al. (8) to calculate the ideal lumbar lordosis (ILL) corresponding to the pelvic anatomy of the patient. According to their formula (LL = 0.54^*^PI + 27.6°) the ILL was 57°. Second, the ILL was adjusted by the patient's age to avoid overcorrection and to decrease surgical invasiveness (4, 9, 10). In the age-group of 65–74-year-old, the threshold of spino- pelvic alignment parameters to avoid significant disability (ODI > 40%) are SVA = 9 cm, PI-LL = 18°, PT = 26°. The threshold values for minimal disability (ODI < 20%) are SVA = 5 cm, PI-LL = 6°, PT = 23°. According to these data (10), target values of SVA between 5 and 9 cm, PI-LL between 6 and 18° and PT between 23 and 25° were determined for the alignment correction. A LL between 37° and 49° corresponded to these parameters, therefore the desired total lordosis correction was 20–32°. Considering all of the surgical issues, and the optimal lordosis distribution, an L1/L2 and L3/L4 transforaminal lumbar interbody fusions (TLIF) and alignment correction by performing an asymmetric pedicle substraction osteotomy (PSO) of about 20° at the L4 level as well as stabilization from Th9 to the iliac bone with posterior fusion was decided as the required surgical intervention to treat the patient.

### Virtual and 3D Printed Models of the Surgery

**Figure 2:**
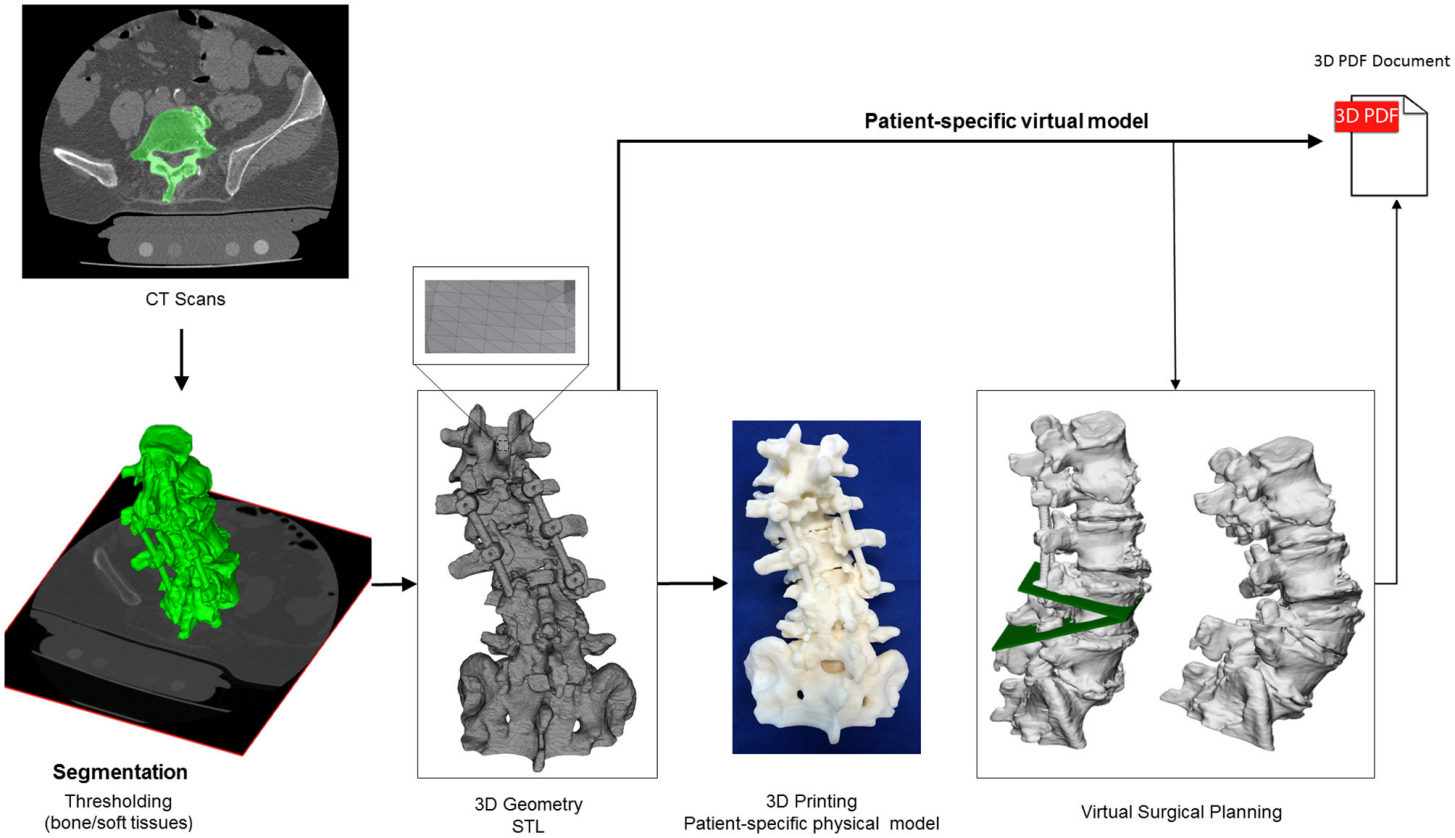
Definition of virtual 3D geometry from CT scan. During the segmentation process the bone volume is first separated from the surrounding soft tissue by thresholding of the greyscale levels of the CT images. The resulting mask (green) voxels represent the 3D volume of the L1-S1 spine segment. Then, from the mask, a triangulated surface mesh is generated in STL format. The STL file serves as an input for 3D printing, with FDM technology. The virtual patient-specific geometry can be edited in CAD software in order to perform virtual surgical intervention (L4 PSO). The virtual geometries are then integrated in the clinical communication as a 3DPDF document.

To better understand the complex anatomical condition at the lower lumbar level, especially in the neuro foraminal and central spinal canal area, patient-specific virtual and physical models were created based on the pre-op CT (Figure 2). The CT data were exported from the hospital PACS in DICOM file format. To comply with the ethical approval and the patient data protection policies, anonymization of the DICOM data was performed using Clinical Trial Processor software (CTP, RSNA, USA) (11). The segmentation process was performed on the 2D CT images (12). The thresholding algorithm and manual segmentation tools (erase, paint, fill etc.) were used in 3D Slicer 4.1.1 free software (Brigham and Women's Hospital, Boston, MA, USA) (13), Figure 2. To evaluate the accuracy of the segmentation process, the Dice Similarity Index (DSI) was calculated (14), obtaining a value of 0.92 and thus providing a highly accurate geometry. Inspection and correction of the 3D geometry was performed with MeshLab 1.3.2 free software (CNR, Pisa, Italy) (15) and universal remeshing with contour preservation was applied. The virtual geometry of the patient's spine [triangulated surface mesh, STereoLithography (STL) format] was printed with a *Fused Deposition Modeling* (FDM) device (Dimension 1200es 3D Printer; Stratasys, Israel; filament type: ABS*plus* in ivory,/scaffold: Soluble Support Technology, SST). In parallel to the printing process, a complex 3D virtual plan was created for the surgery in Autodesk Fusion 360 (Autodesk Inc., California, U.S.A.) Computer Aided Design (CAD) software. First the STL model was converted into a solid body, and then virtually we cut out a wedge shape from the L4 vertebra for an asymmetric 3-column pedicle subtraction osteotomy (PSO) with 20° correction in the sagittal plane. The virtual model and virtual surgical plan was imported in STL format to MeshLab 1.3.2 and subsequently saved as a Universal 3D File (U3D). A 3D Portable Document Format (3DPDF) file, containing the U3D mesh, was created using Adobe Acrobat (version 10 Pro Extended) 3D tools with default activation settings. The 3D visualization parameters were set as follows: CAD optimized lights, white background, solid rendering style, and default 3D conversion settings. The 3DPDF file was then incorporated in the institutional web browser-based SQL database (Oracle Database 12c) as previously described in the literature (16). The document was accessible by clinicians from any institutional desktop PC or mobile device.

### Surgical Treatment and Outcome

**Figure 3:**
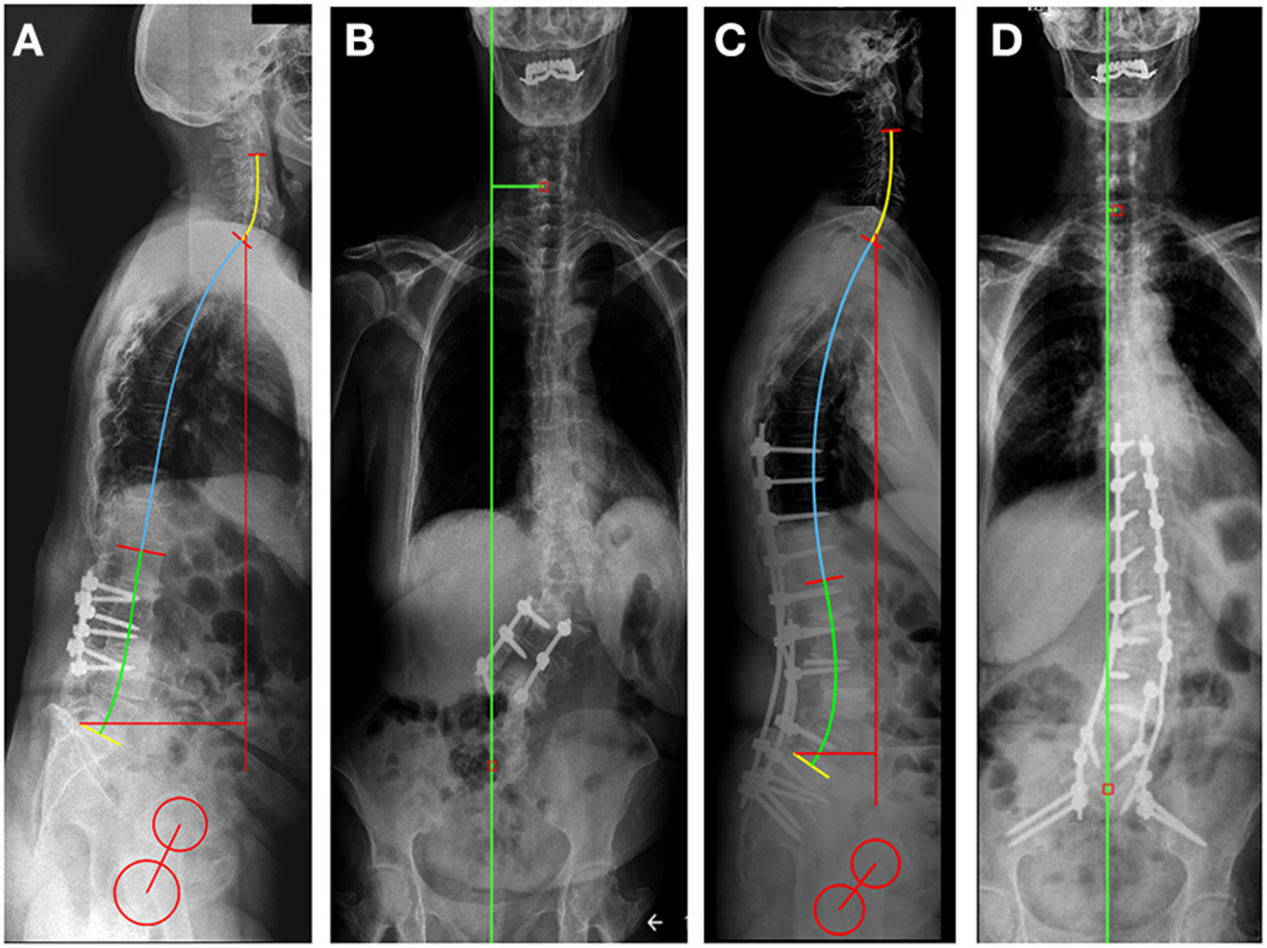
Preoperative **(A,B)** and postoperative **(C,D)** standing X-rays for sagittal **(A,C)** and coronal **(B,D)** spinal alignment evaluation, using the Surgimap software sagittal alignment tools. In the sagittal plane, the SVA was reduced by 7.4 cm compared to the preoperative X-rays due to the Th9-Ileum fixation, correction with the L3-L4 intervertebral fusion (TLIF) and the 3-column osteotomy at the L4 level. The coronal alignment was corrected by reducing the distance between C7 to CSVL from 4.8 cm pre-op **(B)** to 0.5 cm post-op **(D)**.

The surgery was successfully performed without any complications (OR time: 270 min, blood loss: 750 ml). The patient was discharged from the hospital in good condition, 4 days after surgery. 30 degrees of lumbar lordosis correction was achieved, the majority at the L4-S1 levels (17°) (Figure 3, Table 1). The measured change in the sagittal vertical axis (SVA) was 7 cm. In the coronal plane, the C7 to CSVL distance was reduced by 4 cm. The GAP score decreased significantly from 8 to 3. ODI decreased at the 6-months FU to 20 points from 80, the VAS for the LBP decreased to 3 from 9 (17).

## Discussion

Clinical studies about the benefits of new visualization and 3D printing techniques are still very rare worldwide (18). Patient-specific tangible, 3D printed physical models can improve surgical performance and outcome compared to the sole on-screen inspection of the virtual models (19). 3D printed physical models through haptic perception improve understanding of 3D shapes compared to visual perception only (20–22). In a survey based study among the members of AOSpine (23), a high interest among spine surgeons toward the incorporation of 3D technologies (virtual or 3D printed models) into the clinical practice was recorded. The Radiological Society of North America (RSNA) 3D printing Special Interest Group (SIG) published (24) guidelines for medical 3D printing and appropriateness for clinical scenarios. The recommended scenarios do not include the iatrogenic adolescent spinal deformity; although this case demonstrates the benefits.

In a recent systematic review by Lopez et al. (25), in adult spinal deformity, the usage of 3D printing in preoperative planning and in the manufacturing of surgical guides is associated with increased screw accuracy and favorable deformity correction outcomes. In our study, the physical model not only provided guidance in the preoperative planning phase, but also aided the surgeon in understanding the complex anatomy during the surgery.

It is challenging in the surgical management of adult spinal deformity to determine the degree of planned correction, particularly in patients with severe preoperative malalignment. Less aggressive correction may constitute a reasonable compromise between radiographic alignment goals and perioperative and postoperative risk (9). The Surgimap software allowed the measurement of pre- and postoperative X-rays with ease and speed, providing a vast array of opportunities for assessment of spinal deformity and surgical planning. The aid of 3D virtual and 3D printed models, and X-ray based planning software allowed us to achieve a LL of 47° after the surgery providing the restoration of global balance showed by the improvement of the GAP score. The well-planed surgical correction of the lumbar alignment provided the restoration of the global spinopelvic balance, resulting in the reduction in pain and disability as well as improvement in health-related quality of life. The improved global parameter (GAP score of 3) corresponds to a moderately disproportioned alignment with low chance of postoperative mechanical complication (6).

The limitation of the described approach is that currently it is uncommon for medical centers to have access to a 3D printing facility or lack the know-how for image processing needed for model preparation. The time needed for the presented visualization, printing, and planning is also a limitation as it is not always available before surgery.

## Conclusion

A patient-specific 3D virtual and printed physical geometry as well as computer-aided surgical planning were used to develop the optimal surgical plan for the deformity correction in a complicated iatrogenic adult spinel deformity case. The surgery was successfully implemented providing the planned correction of the lumbar alignment. The printed physical model was considered advantageous by the surgical team in the pre-surgical phase and during the surgery as well. The chosen FDM technology provided an accurate, robust, and affordable physical model. The model not only clarifies the geometrical problems, but it can also improve the outcome of the surgery by preventing complications and reducing surgical time.

## Data Availability Statement

The original contributions presented in the study are included in the article/Supplementary Material, further inquiries can be directed to the corresponding author.

## Ethics Statement

The study was approved by the National Ethics Committee of Hungary, the National Institute of Pharmacy and Nutrition (Reference Number: ETT TUKEB IV/6329-1/2020/EKU). Informed consent was obtained from the participant. The patients/participants provided their written informed consent to participate in this study. Written informed consent was obtained from the individual(s) for the publication of any potentially identifiable images or data included in this article.

## Author Contributions

PE and AL contributed to the conception and design of the study. PE, JF, FB, MT, and BH did the CT based image analyses which provided the 3D visualizations, the virtual model, and the CAD design. PE, JF, and AL performed the pre and postoperative X-ray measurements. MB and MT provided the 3D print. AL, AB, and PE performed the surgery described in this case report. PE and JF wrote the first draft of the manuscript and prepared the pictures together with AL. All authors contributed to manuscript revision, read, and approved the submitted version.

## Conflict of Interest

MB was employed by the company Do3D Innovations Ltd. The remaining authors declare that the research was conducted in the absence of any commercial or financial relationships that could be construed as a potential conflict of interest.
