# Supplementary material for: Complicated Postoperative Flat Back Deformity Correction With the Aid of Virtual and 3D Printed Anatomical Models: Case Report
Source: Front Surg. 2021 May 28;8:662919. doi: 10.3389/fsurg.2021.662919 (PMC8192795; doi:10.3389/fsurg.2021.662919)
Supplement: Supplementary file 1 [file Data_Sheet_1.pdf]

Supplementary Materials

Complicated postoperative flat back deformity correction with the aid of virtual and 3D printed anatomical models: case report

Jennifer Fayad[1,2,3], Mate Turbucz[1,4], Benjamin Hajnal[1], Ferenc Bereczki[1,4], Marton Bartos[5], Andras Bank[6], Aron Lazary[3,6*], Peter Endre Eltes[1,3*]

1. In Silico Biomechanics Laboratory, National Center for Spinal Disorders, Buda Health Center, Budapest, Hungary
2. Department of Industrial Engineering, Alma Mater Studiorum, Universita di Bologna, Bologna, Italy
3. Department of Spine Surgery, Semmelweis University, Budapest, Hungary
4. School of PhD Studies, Semmelweis University, Budapest, Hungary
5. Do3D Innovations Ltd., Budapest, Hungary
6. National Center for Spinal Disorders, Buda Health Center, Budapest, Hungary

*authors contributed equally to the work

Aron Lazary, corresponding author

National Center for Spinal Disorders, Királyhágó St. 1-3, Budapest 1126, Hungary

Tel.:(36) 1-887-7900, Fax.: (36) 1-887-7987, Email address: aron.lazary@bhc.hu

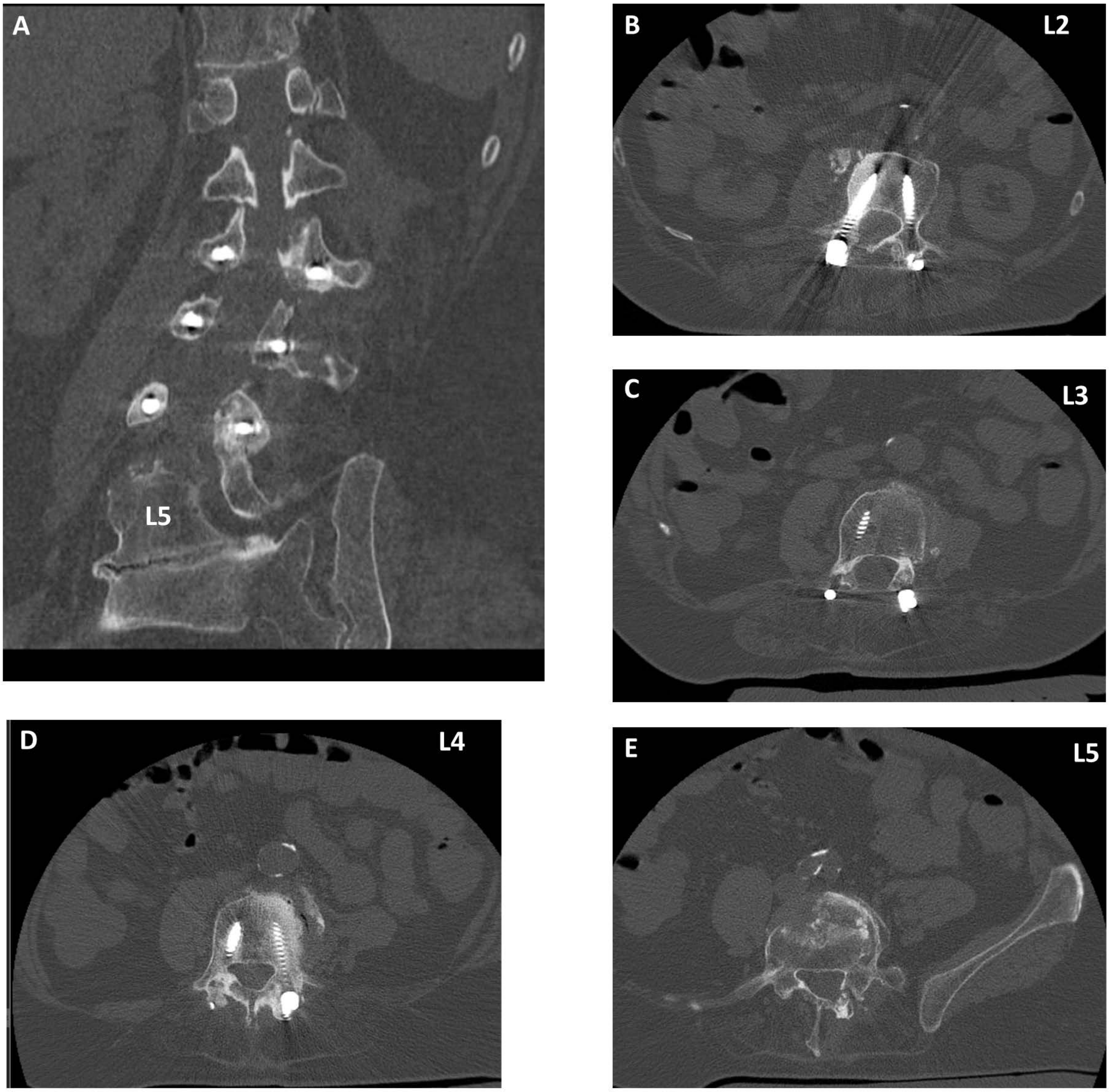

Supplementary Figure 1. Preoperative computed tomography (CT) images of the lumbar spine. **A** coronal CT scan image at the pedicle level (L2, L3, L4), L5 vertebral body is marked as a landmark. **B** axial CT scan image at the level of the L2 pedicle. **C** axial CT scan image at the level of the L3 pedicle. **D** axial CT scan image at the level of the L4 pedicle. **E** axial CT scan image at the level of the L5 pedicle**.**

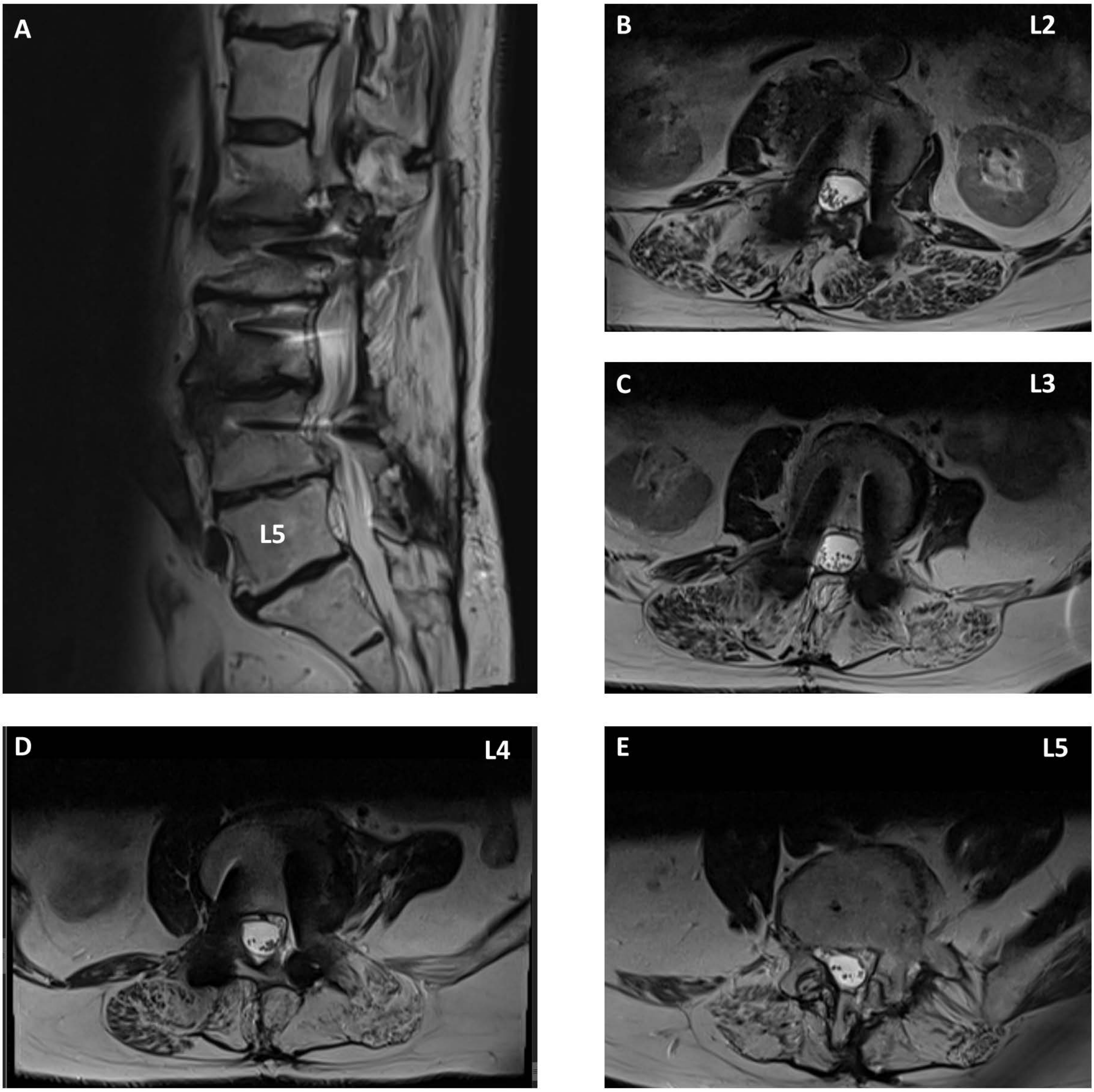

Supplementary Figure 2. Preoperative magnetic resonance imaging (MRI) of the lumbar spine. **A** sagittal MRI scan image at the pedicle level (L2, L3, L4), L5 vertebral body is marked as a landmark. **B** axial MRI scan image at the level of the L2 pedicle. **C** axial MRI scan image at the level of the L3 pedicle. **D** axial MRI scan image at the level of the L4 pedicle. **E** axial MRI scan image at the level of the L5 pedicle**.**
